# Psychological Mechanisms Linking County-Level Income Inequality to Happiness in China

**DOI:** 10.3390/ijerph15122667

**Published:** 2018-11-27

**Authors:** Jiaoli Cai, Li Zhang, Yulin Zhao, Peter C. Coyte

**Affiliations:** 1 School of Economics and Management, Beijing Jiaotong University, No.3 Shangyuancun, Haidian District, Beijing 100044, China; lzhang@bjtu.edu.cn; 2 School of Economics, Wuhan University of Technology, 122 Luoshi Road, Wuhan 430070, China; ylz1115@163.com; 3 Institute of Health Policy, Management and Evaluation, University of Toronto, Health Sciences Building, 155 College Street, Suite 425, Toronto, ON M5T 3M6, Canada; peter.coyte@utoronto.ca; 4 Canadian Centre for Health Economics, 155 College Street, Toronto, ON M5T 3M6, Canada

**Keywords:** happiness, income inequality, trust, fairness, China

## Abstract

*Background* In China, income levels and living standards have improved significantly, but many Chinese citizens still do not feel any happier. This phenomenon may be attributed to increased income inequality. *Methods* Using data from the 2013 Chinese General Social Survey (CGSS), we employed multilevel structural equation modeling (MSEM) to investigate the impact of county-level income inequality on individual-level happiness in China and multilevel mediation analysis with structural equation modeling (MMSEM) to explore the mechanisms through which income inequality impacted happiness. *Results* A negative relationship between income inequality and happiness was found. The negative association between them was explained by two psychological mechanisms, i.e., fairness and trust. The findings explained a “Chinese puzzle,” i.e., why people do not feel happier despite improved income and living standards. *Conclusions* Our findings may provide a reference for policy makers to implement policies designed to improve individual happiness. What is important now is to reduce income inequality, and to potentially improve perceptions of fairness and trust in China.

## 1. Introduction

In the past decade, profound changes have taken place in the Chinese economy and society due to economic reforms and dramatic economic growth. With these changes, income levels and living standards have improved significantly, but many Chinese citizens still do not feel any happier [1]. Possible explanations for this “Chinese puzzle” may be that during the process of economic development, income inequality has increased, thereby reducing people’s overall happiness. For example, in China, happiness declined from 1990 to 2000 in spite of great improvements in material living standards, and this trend may be attributed to increased income inequality [2].

Actually, income inequality has become one of the greatest challenges for policy decision-makers in China. Since 2003, the Gini coefficient, which is an income inequality measure, continued to increase, reaching 0.491 by 2008 [3]. Income inequality in China was higher than all OECD countries (Mexico was the highest there and this was just above its Gini). Since 2008, the Gini coefficient has been quite uniform, and remains alarmingly higher than the international warning line of 0.40 [3]. According to a study by Xie and Zhou [4], income inequality in China has been at very high levels since 2005, with the Gini coefficient rising from 0.53 to 0.55. Income inequality is much higher than what has been acknowledged in the government statistics [4]. Increasing income inequality may lead to many social problems, such as crime rates, violence, and homicide [5].

The relationship between income inequality and happiness has attracted much attention in the social science literature. However, because the results were mixed in previous studies, knowledge about this relationship is still inconclusive [6,7]. While a negative relationship between income inequality and happiness has generally been reported [8,9,10], there are a few studies that have found a positive relationship [11,12]. Other studies have shown that there was no relationship between income inequality and happiness [13]. In the Chinese context, there have been studies that have explored the relationship between income inequality and happiness [1,2,14,15,16,17]. For example, Easterlin et al. in the World Happiness Report 2017 chapter found a U-shaped subjective well-being (SWB) since 1990s in China, i.e., SWB experienced a downward trend in 1990s but has also increased since 2005 [18]. They also indicated that income inequality as measured by the Gini coefficient has increased when SWB is both falling and rising. Therefore, the changes in income inequality could not solely explain the U-shaped movement of SWB. Jiang et al. found a positive association between income inequality and happiness [1]. Knight et al. found a negative association between income inequality and happiness [14]. Again, while the overall literature is inconclusive, most of these studies report a negative association between income inequality and happiness.

What is more, although there were many studies on the relationship between income inequality and happiness, little empirical research has been conducted to explore the mechanisms of influence, especially in low- to middle-income countries. One study conducted in the United States, a high-income country, explored the relationship between national-level income inequality and happiness [19]. Another British study by Wilkinson and Pickett found that inequality is associated with lower well-being of various kinds through increased social tensions [20]. China has different national conditions from the United States and the United Kingdom, which limits the generalizability of these findings to China. We believe that it is necessary to explore the mechanisms by which income inequality impacts happiness in the Chinese context. To our knowledge, there is a paucity of research exploring these mechanisms, and from our review of the literature, we believe our study is the first to assess these transmission mechanisms for China. Moreover, modern China represents an important case study because it has experienced fast economic growth over the last decade, and it has also witnessed dramatic growth in income inequality. Previous studies conducted in China often used data from 2005 or earlier, which may not reflect the relationship between income inequality and happiness in more recent years. As such, a reassessment of the empirical relationship between happiness and income inequality in China using data from the modern era may better reflect the state of happiness in modern China.

The purposes of this study were to assess whether income inequality impacted individual happiness in modern China, and to explore the mechanisms through which income inequality affected happiness, by using micro-level data obtained from the 2013 Chinese General Social Survey (CGSS).

## 2. Three Hypotheses

Income inequality in China has reached very high levels since 2005 [4]. Income inequality seems to persist, and social and income mobility continue to decline in modern China. It is difficult for people at the bottom to move to the upper class [21]. When there is no hope for upward mobility of income and social class, people will have an aversion to income inequality. Income inequality may lead to frictions and conflicts among different groups, thus reducing happiness. Income inequality may make some people believe that they are at the bottom of society and cause psychological dissatisfaction. Thus, income inequality may lead to a negative impact on individual happiness. Based on this, we hypothesized that the income inequality is negatively related to happiness.

(1) H1: Income inequality has a negative impact on happiness.

In this study, we explored two psychological mechanisms to account for the link between income inequality and happiness following Oishi et al. [19]. One is through a fairness mechanism, while the other is through a mechanism of trust. Perceived fairness has been shown to be an important factor that affects happiness [22]. The fairness mechanism comes from equity theory. Equity theory, proposed by Adams [23], is about whether the resource distribution is fair to both relational partners. Based on this theory, stress will be suffered by both the person who is under-rewarded and the person who is over-rewarded. This stress makes them strive to restore a fair relationship [24]. Equity theory maintains that balanced relationships contribute to higher levels of well-being [25]. However, when people find themselves involved in unfair relationships, they may become distressed. The greater the inequity the individual perceives (in the form of either overreward or underreward), the more distress the individual feels [24]. People who get too much may feel guilty or shameful. People who get too little may feel angry or humiliated [24]. Though both people who get too much and people who get too little feel distressed, for people who get too little, the feeling of unfairness can be stronger [26]. When people find themselves at a disadvantage in comparison with other people, these people will experience a sense of unfairness, thereby stimulating negative psychological perceptions [27]. The unbalanced economic development during the social and economic transition period has led to the widening income inequality between the rich and the poor in China. Some groups may have not been able to share in the fruits of social progress or have been in the position of economic disadvantage. Thus, they feel a strong sense of frustration, relative deprivation, and unfairness. Based on equity theory and China’s reality, we hypothesized that unfairness may explain the negative relationship between income inequality and happiness.

(2) H2: Fairness mechanism may account for the association between income inequality and happiness.

Trust is a core component of social capital. Trust can promote economic growth and the development of finance by decreasing transaction costs and promoting investment [28,29]. Both economic theory and empirical studies suggest that trust impacts cooperative behaviors [30]. Trust has been shown to be an important predictor of happiness [31,32,33]. In a country with a high level of trust, individuals can easily get social support [34], where social support, especially emotional support, is an important factor in the promotion of psychological well-being [35]. Family emotional support is positively related to happiness. [36]. However, once income inequality increases, trust between people will be eroded [19,37]. High inequality strengthens the differences of status, which can easily lead to interpersonal estrangement and mistrust among people [37], and finally may cause unhappiness. In China, effects to modify the income distribution have not yet reduced income inequality. For the groups at the bottom of society, their interests have not been achieved, thus exacerbating the conflicts of interest and tensions between people. The lack of trust caused by such interpersonal tensions may inspire personal dissatisfaction, which subsequently lowers happiness. Therefore, we hypothesized that the lack of trust caused by income inequality may reduce happiness.

(3) H3: Trust may be a mechanism through which income inequality is linked with happiness.

**Figure 1:**
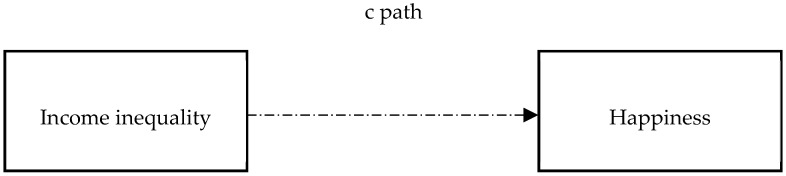
Conceptual model examining Hypothesis 1.

**Figure 2:**
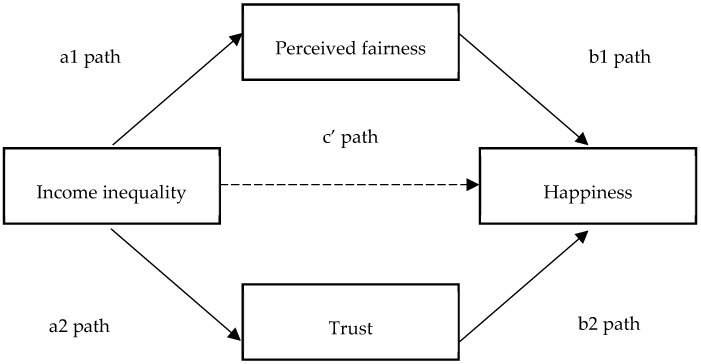
Conceptual model examining Hypothesis 2.

The conceptual depiction explaining the impact of income inequality on happiness is shown in Figure 1 (see Figure 1). The conceptual depiction explaining the hypothesized links between income inequality, fairness, trust, and happiness is shown in Figure 2 (see Figure 2).

The path c represents the total effect of income inequality on happiness (absence of mediators). The product of path a1 and b1 represents the indirect effect of income inequality on happiness, being transmitted through fairness. The product of path a2 and b2 represents the indirect effect of income inequality on happiness, being transmitted through trust. Path c’ represents direct effect of income inequality on happiness.

The paper is structured as follows. The next section presents the data and method, followed by the results. Then discussion and conclusions are given in the last section.

## 3. Data and Method

### 3.1. Data

The data in this study were drawn from the 2013 Chinese General Social Survey (CGSS), which was organized by the Department of Sociology at Renmin University of China and the Survey Research Center of Hong Kong University of Science and Technology in September and October, 2013. This survey was designed to understand the social structure in China and the quality of life for Chinese rural and urban families. The survey used a five-stage stratified sampling method (province, county, town, village, and household), covers 28 provinces (municipalities) (There were 31 provinces, municipalities, and autonomous regions in mainland China. This survey covered 28 provinces, municipalities, and autonomous regions except Xinjiang, Tibet, and Hainan), 134 counties (districts), 374 towns (streets), 491 villages (neighborhood committees), and 11,438 households, thereby yielding 11,438 samples, providing a representative sample for China. Respondents who were Chinese nationals and who were aged 18 years or older were chosen. The CGSS contained detailed information on individual characteristics (such as sex, age, marriage, education, and income), household characteristics (such as household size and place of residence), and other variables (such as happiness). By sorting data and deleting the missing data, almost 81% (or 9239) of the survey respondents were included in our analysis.

### 3.2. Measures

#### 3.2.1. Happiness

The dependent variable in the current study was derived as a happiness score from responses by survey respondents. Respondents to the survey were asked to answer the question, “In general, do you think your life is happy or not?” Respondents chose an option on a five-point scale comprising “1 = not happy at all,” “2 = not happy,” “3 = so-so,” “4 = happy,” and “5 = very happy.” Happiness indicators have been used in several studies [19,38,39,40,41,42]. In this study, the happiness variable was treated as a continuous variable following Wang et al. [17], with higher scores indicating more happiness. We selected this scoring scheme as it is intuitive and helps in the interpretation of our findings when we used multilevel mediation analysis. What is more, the estimates from our ordered probit/logit model were similar to the results from the ordinary least squares (OLS) model [43,44].

#### 3.2.2. Income Inequality

The income we used to calculate inequality was the per capita household income [45]. Household income included wage, household gardening income, farming income, livestock income, fishing income, business income, housing subsidies, child care subsidies, and other types of subsidies income. The Gini coefficient was used in our study to capture county-level income inequality. We chose county-level income inequality because people may be more inclined to compare with the people around them. The Gini coefficient is one of the most widely used inequality measures and it ranges from 0 to 1 [46]. A Gini value of 0 indicates that income was distributed equally among the population, while a Gini value of 1 is indicative of the greatest possible degree of income inequality. For each county, we calculated a Gini coefficient based on individual income. Individuals in the same county had a common county-level Gini coefficient. Because there were 134 counties in our analysis, we derived 134 separate Gini coefficients for use in our analysis.

*Perceived fairness and trust*. Perceived fairness was used to test for the fairness mechanism. Survey respondents were asked “In general, do you think that the current society is fair or not?” Answers from 1 to 5 corresponded to the degree of fairness, from “totally unfair” to “totally fair.” Fairness was regarded as a continuous variable, which means that higher scores were indicative of higher perceived degrees of fairness. Trust was used to test for the trust mechanism. Survey respondents were asked “In general, do you agree that in this society, most people are trustworthy?” The answers were “1 = strongly disagree,” “2 = disagree,” “3 = so-so,” “4 = agree,” and “5 = strongly agree.” We treated this variable as a continuous variable, with values ranging from 1 to 5, thereby representing a scale from the “lowest level of trust” to the “highest level of trust.” The degree of trust was higher the greater the score.

#### 3.2.3. Other Control Variables

Based on the previous studies [22,47,48], we controlled for individual and household characteristics, such as sex, age, age squared, marital status, ethnicity, household size, residence, and work status. We also controlled for political status (party member or not) as party membership in China offers individuals greater opportunities to express their views than others, thus affecting happiness [49]. The level of education and perceived health were also included in our analysis. Subjective well-being increased with years of schooling [50]. Health was also a potential factor that may affect happiness [50,51,52]. The level of education was divided into three levels comprising primary education, secondary education, and tertiary education. Health was operationalized as a categorical variable and took on one of three categories—“good,” “fair,” or “poor”—based on a respondent’s evaluation of their perceived health status. Income has been shown to be associated with happiness [10,53]. Individual income was last year’s annual income and it was log-transformed in our empirical work to allow for a non-linear relationship between happiness and income, as suggested by previous work [17]. We also controlled for the region of residence through use of regional dummy variables. According to China’s economic development and administrative divisions, 28 provinces were divided into three categories, namely Eastern China (including 10 provinces). (Eastern region included 10 provinces: Beijing, Tianjin, Hebei, Shandong, Jiangsu, Shanghai, Zhejiang, Fujian, Guangdong and Liaoning), Middle China (including 8 provinces) (Middle region included 8 provinces: Shanxi, Henan, Hubei, Hunan, Jiangxi, Anhui, Jilin and Heilongjiang), and Western China (including 10 provinces) (Western region included 10 provinces: Chongqing, Sichuan, Guangxi, Guizhou, Yunnan, Shanxi, Gansu, Neimenggu, Ningxia, Qinghai).

### 3.3. Statistical Analysis

A multilevel structural equation modeling (MSEM) was used to explore the relationship between county-level income inequality and individual-level happiness. Structural equation modeling encompasses a broad array of models from linear regression to measurement models to simultaneous equations. Actually, regarding our analysis, the results from structural equation modeling were the same with the results from linear regression. However, when analyzing the mechanisms, the SEM was better because it could reflect the indirect effect. Therefore, the SEM was used. A multilevel mediation analysis with structural equation modeling (MMSEM) was used to explore the psychological mechanisms through which income inequality impacts happiness. We used multilevel models, because income inequality was measured at the county level, while happiness was measured at the individual level. Specifically, first, we examined the relationship between county-level income inequality and individual-level happiness—Hypothesis 1. Second, we assess the psychological mechanisms that account for the relationship between county-level income inequality and individual-level happiness—Hypotheses 2 and 3. All the analyses were conducted using Stata version 13.0 for Mac (StataCorp LP, College Station, TX, USA).

## 4. Results

### 4.1. Descriptive Analysis

Table 1 presents the descriptive statistics for the study variables (see Table 1). The mean score for happiness was 3.77. The average Gini coefficient at the county-level was 0.44. Per capita annual income for respondents was 24,694.60 Yuan (USD 1.00 = CNY 6.07). The sample was equally divided between men and women (51.81% vs 48.19%). Survey respondents were mostly of Han nationality (91.78%), married (80.95%), and from urban areas (60.99%). Almost half (49.24%) of the respondents received secondary education and 43.06% of the respondents were engaged in non-farm work. Most of the respondents reported good health (65.12%).

### 4.2. Regression Results

**Figure 3:**
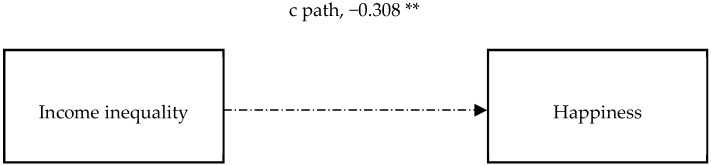
The impact of income inequality on happiness.

**Figure 4:**
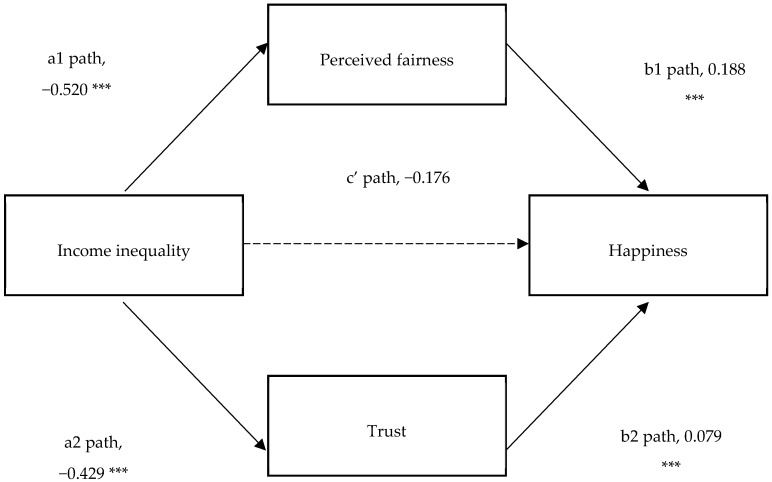
The links between income inequality and happiness.

Table 2 shows the MSEM regression results (see Table 2). After holding other control variables constant, county-level income inequality was negatively associated with individual-level happiness, supporting H1. Table 3 shows the results of mediation analysis (see Table 3) where the two key psychological mechanisms were controlled. Income inequality as measured using the Gini coefficient was negatively associated with perceived fairness, while perceived fairness was positively associated with happiness. This finding suggests that an increase in income inequality reduces perceived fairness, which subsequently lowers happiness. Income inequality had a significant and negative impact on trust, while trust had a significantly positive effect on happiness. This finding suggests that an increase in income inequality reduces trust, which subsequently lowers happiness. When perceived fairness and trust were included in the regression together, the coefficient on county-level inequality became insignificant, meaning that the direct effect of county-level income inequality on individual-level happiness was eliminated through the inclusion of both fairness and trust. Figure 3 and Figure 4 intuitively show the relationship between the variables (see Figure 3 and Figure 4). Path c shows the total effect of income inequality on happiness. The total effect was significant at the 5% significance level. The product of path a1 and path b1 shows the indirect effect of county-level income inequality on individual-level happiness, being transmitted through perceived fairness. The indirect effect through perceived fairness was a1 × b1 = −0.098, and the indirect effect was significant. The product of path a2 and path b2 shows the indirect effect of county-level income inequality on individual-level happiness, being transmitted through trust. The indirect effect through trust was a2 × b2 = −0.034, and the indirect effect was also significant. Path c’ shows the direct effect of income inequality on happiness, but the direct effect was eliminated after controlling for perceived fairness and trust. The total significant portion of the indirect effect on happiness via fairness and trust was −0.132 (a1 × b1 + a2 × b2 = −0.132), whose absolute value (0.132) was smaller than the absolute value of total effect of income inequality on happiness (0.308). This indicates that though the relationship between income inequality and happiness has been mediated by fairness and trust, there may exist other additional mediators. Comparing these two mechanisms, it seems that the fairness mechanism accounts more for the negative association between income inequality and happiness. Because the indirect effect through perceived fairness was −0.098, whose absolute value is higher than the absolute value of indirect effect on happiness via trust. This indicates that reducing the income inequality and rebuilding the fairness mechanism are more important measures to improve happiness.

We removed counties that contained no more than 10 observations to conduct analysis and found that the results were robust. Similarly, we removed counties that contained no more than 20 observations to conduct the robustness test, and found the results were consistent. The results of the robustness tests were presented in the Supplementary materials Tables S1–S4.

We also divided the sample into sub-samples by income level, residence, gender, and age group to conduct analyses. The results of the sub-sample analyses are reported in the Appendix A in order to save space. The results were robust. All the results showed a negative correlation between income inequality and happiness. The mechanisms of fairness and trust were revealed again. However, for different groups, the two mechanisms played a slightly different role. For poor people, it seems that the fairness mechanism accounts more for the negative association between income inequality and happiness; while for rich people, a trust mechanism seems to explain more regarding the negative association between income inequality and happiness. The indirect effect through perceived fairness was −0.534 × 0.212 = −0.113 and −0.308 × 0.137 = −0.042 for poor people and rich people, respectively (Table A1, Table A2, Table A3 and Table A4). The indirect effect through trust was −0.409 × 0.074 = −0.030 and −0.687 × 0.090 = −0.062 for poor people and rich people, respectively (Table A1, Table A2, Table A3 and Table A4). Similarly, for urban people, a trust mechanism seems to explain more about the relationship between income inequality and happiness (Table A5 and Table A6); while for rural people, a fairness mechanism seems to explain more about the relationship (Table A7 and Table A8). For young people, a fairness mechanism seems to explain more about the relationship between income inequality and happiness (Table A9 and Table A10); while for old people, a trust mechanism explains more about the relationship (Table A11 and Table A12). Whether for men or women, fairness seems to account more for the association between income inequality and happiness (Table A13, Table A14, Table A15 and Table A16).

## 5. Discussion

This study examined the impact of income inequality on happiness in China and explored the mechanisms through which income inequality impacted happiness. We found that income inequality had a negative impact on happiness, and we also found that fairness and trust mechanisms accounted for the negative association between income inequality and happiness in China.

Our findings that income inequality negatively impacted happiness are in line with previous studies conducted in China [2,16,54]. Brockmann et al. [2] found that over the decade from 1990 to 2000, income inequality in China became increasingly skewed towards the upper income strata, and income inequality was an increasingly important factor in lowering happiness. Smyth and Qian [16] examined the relationship between inequality and happiness in urban China using a large-scale survey administered in 31 cities in September 2002. They found that those who perceived the income distribution to be unequal reported lower levels of happiness. Wu and Li [54], using data from a national representative survey conducted in 2005, examined the subjective consequence of rising income inequality amidst rapid economic growth in China. They found that income inequality had a negative effect on an individual’s life satisfaction. Our findings contrast with the finding by Wang et al., 2015 [17], who found an inverted U-shaped relationship between income inequality and happiness by using data from the 2005 CGSS. They found that individual happiness increased with inequality when the Gini coefficient was less than 0.405, and fell with inequality for larger values of the Gini coefficient. They used the “tunnel effect” to explain their results, i.e., they reported that at lower levels of income inequality, inequality signaled potential future income opportunities, but once inequality exceeded a threshold, individuals would become discouraged. In our study, we tried to control for the potential non-linear relationship between inequality and happiness through inclusion of squared values of the Gini but found the coefficient on Gini squared was insignificant. Our results do not provide evidence in support of the “tunnel effect” for modern China. As we showed in Section 2, income inequality between different groups in China was persistent and high since 2005, and it seems difficult for people at the bottom to enter into the upper class. People are disappointed with the slow income mobility and social mobility [21]. Thus, income inequality as an unpleasant phenomenon may cause unhappiness. Our findings contrast with the findings by Clark [11] and Tomes [12], which do not support the hypothesis that there exists a negative association between income inequality and happiness. However, the two studies are either very obscure (the one done in 2003) or very old before the relationship became established.

We also found that two psychological mechanisms may explain why income inequality has caused unhappiness in China, which is consistent with one study conducted in the United States by Oishi et al. [19]. Oishi et al. [19] examined the relationship between income inequality and happiness. They used national-level income inequality and found a negative association between national-level income inequality and individual-level happiness. They explained that Americans perceived others to be less fair and less trustworthy in times of income inequality than in times of income equality. We found evidence for similar mechanisms in our study, though there are different national conditions between the United States and China. The lack of fairness and trust due to income inequality was evident in China. In China’s social and economic reform, along with the break of the traditional mode of egalitarian income distribution, China set up a new distribution mode that allows some people and regions to get rich first, when and where conditions permit. Persons and regions with faster economic development can help promote the progress of persons and regions with slower development [17]. However, some persons and regions become rich while some are still poor. What is more, the income inequality between the rich and the poor are becoming larger and larger [55,56]. The huge income inequality may cause a strong sense of unfairness, and may lead to crime rates, violence, mistrust, and homicide rates [5], thus causing unhappiness. For poor people, rural residents, and young people, fairness accounts more for the association between income inequality and happiness. For rich people, urban residents, and old people, trust accounts more for the relationship.

It was important to recognize the limitations of our study. First, the data we used were not longitudinal, so we were not able to analyze happiness over time. However, we used data for 2013, which could reflect individual happiness in modern China. Second, happiness, perceived fairness, and trust variables were each measured using single items. Although the same single-item happiness, perceived fairness, and trust measures have often been used [57,58], it is important to use multi-item scales to verify the current findings. Third, though we found that the significant direct effect of income inequality on happiness (c’) disappeared after fairness and trust mechanisms were included, we could not conclude that the association between income inequality and happiness was completely mediated by the two mechanisms because a nonsignificant direct effect (c’) should not be viewed as a stopping rule in the search for additional mediators, i.e., though one or several proposed mediator(s) fully accounts for an effect, there may still exist other mediators [59]. Our results showing that the significant portion of the indirect effect via fairness and trust was smaller than the total effect also show that the two mechanisms were not the only mechanisms. Therefore, other mechanisms should be explored in the future. Fourth, our data were derived from China, where there exists huge income inequality. These findings may not be generalizable to other countries with low income inequality. However, the findings may be useful for countries with similar backgrounds.

## 6. Conclusions

In conclusion, the findings have several implications for the design and development of public policy to promote happiness. First, this study provides empirical evidence to support the negative association between income inequality and happiness. Unfairness and mistrust caused by income inequality were identified as the key mechanisms by which individuals in China experienced unhappiness. Therefore, what is important now is to reduce income inequality, and to potentially improve perceptions of fairness and trust in China. What is more, rebuilding the fairness mechanism is more important to improve happiness. Lastly, for different groups of people, targeted specific measures should be taken to improve their happiness.

## Figures and Tables

**Table 1:** Descriptive statistics (*n* = 9239).

Continuous Variables	Mean	SD	Min	Max
Happiness	3.77	0.83	1.00	5.00
Gini coefficient	0.44	0.11	0.16	0.68
Individual income (CNY)	24,694.60	37,829.09	80.00	1000,000.00
Household size	3.05	1.38	1.00	12.00
Trust	3.30	1.03	1.00	5.00
Perceived fairness	3.00	1.04	1.00	5.00
Age	49.83	15.80	18.00	98.00
Age squared/100	27.33	16.42	3.24	96.04
Categorical variables	Percentage			
Sex				
Male	51.81			
Female	48.19			Reference
Ethnicity				
Han	91.78			
Minority	8.22			Reference
Political status				
Party member	14.67			
Non-members	85.33			Reference
Marital status				
Married	80.95			
Single	8.40			
Others (divorced, widowed, separated)	10.65			Reference
Residence				
Urban	60.99			
Rural	39.01			Reference
Work Status				
Non-farm work	43.06			
Farm work	22.78			
Not working	34.16			Reference
Education degree				
Primary education	34.55			Reference
Secondary education	49.24			
Tertiary education	16.21			
Health status				
Good	65.12			
Fair	19.03			
Poor	15.86			Reference
Regions				
East	41.11			
Middle	33.49			
West	25.40			Reference

SD: Standard Deviation.

**Table 2:** The impact of income inequality on happiness.

Variables	Happiness
Gini	−0.308 **
	(0.140)
Income (CNY)	0.136 ***
	(0.010)
Sex (1 = Male)	−0.097 ***
	(0.017)
Age	−0.030 ***
	(0.004)
Age squared/100	0.035 ***
	(0.003)
Ethnicity (1 = Han)	−0.073 **
	(0.031)
Political status (1 = Party member)	0.086 ***
	(0.026)
Marital Status (Ref: Others)	
Married	0.233 ***
	(0.029)
Single	−0.009
	(0.045)
Household size	0.049 ***
	(0.007)
Residence (1 = Urban)	−0.100 ***
	(0.022)
Work Status (Ref: Not working)	
Non-farm work	−0.017
	(0.023)
Farm work	−0.002
	(0.027)
Education degree (Ref: Primary education)	
Secondary education	0.026
	(0.022)
Tertiary education	0.060 *
	(0.033)
Health Status (Ref: Poor)	
Good	0.422 ***
	(0.025)
Fair	0.186 ***
	(0.029)
Regions (Ref: West)	
East	0.021
	(0.024)
Middle	−0.048 **
	(0.022)
Constant	2.245 ***
	(0.148)
Observations	9239
Log likelihood	−138,677.26

Note: Standard errors in parentheses; *** *p* < 0.01, ** *p* < 0.05, * *p* < 0.1.

**Table 3:** Mediation analysis.

Variables	Mediators		Dependent Variable
	Perceived Fairness	Trust	Happiness
Gini	−0.520 ***	−0.429 ***	−0.176
	(0.105)	(0.106)	(0.140)
Perceived Fairness			0.188 ***
			(0.008)
Trust			0.079 ***
			(0.008)
Income (CNY)			0.129 ***
			(0.009)
Sex (1 = Male)			−0.087 ***
			(0.017)
Age			−0.029 ***
			(0.003)
Age squared/100			0.031 ***
			(0.003)
Ethnicity (1 = Han)			−0.061 **
			(0.029)
Political status (1 = Party member)			0.070 ***
			(0.024)
Marital Status (Ref: Others)			
Married			0.238 ***
			(0.028)
Single			−0.003
			(0.043)
Household size			0.049 ***
			(0.006)
Residence (1 = Urban)			−0.033
			(0.021)
Work Status (Ref: Not working)			
Non-farm work			−0.028
			(0.022)
Farm work			−0.035
			(0.026)
Education degree (Ref: Primary education)			
Secondary education			0.041 **
			(0.021)
Tertiary education			0.048
			(0.032)
Health Status (Ref: Poor)			
Good			0.379 ***
			(0.024)
Fair			0.177***
			(0.027)
Regions (Ref: West)			
East			0.049 **
			(0.023)
Middle			−0.025
			(0.021)
Constant	2.819 ***	3.073 ***	1.473 ***
	(0.047)	(0.046)	(0.140)
Observations			9239
Log likelihood			−165159.14

Note: Standard errors in parentheses; *** *p* < 0.01, ** *p* < 0.05.

## Supplementary Materials

The following are available online at http://www.mdpi.com/1660-4601/15/12/2667/s1, Table S1: The impact of income inequality on happiness, Table S2: Mediation analysis, Table S3: The impact of income inequality on happiness, Table S4: Mediation analysis.

## Appendix A

**Table A1:** The impact of income inequality on happiness (poor people).

Variables	Happiness
Gini	−0.358 ***
	(0.144)
Income (CNY)	0.149 ***
	(0.014)
Sex (1 = Male)	−0.102 ***
	(0.022)
Age	−0.031 ***
	(0.005)
Age squared/100	0.036 ***
	(0.004)
Ethnicity (1 = Han)	−0.075 **
	(0.036)
Political status (1 = Party member)	0.104 ***
	(0.037)
Marital Status (Ref: Others)	
Married	0.240 ***
	(0.037)
Single	0.007
	(0.062)
Household size	0.041 ***
	(0.008)
Residence (1 = Urban)	−0.116 ***
	(0.026)
Work Status (Ref: Not working)	
Non-farm work	−0.026
	(0.029)
Farm work	−0.001
	(0.031)
Education degree (Ref: Primary education)	
Secondary education	0.041
	(0.026)
Tertiary education	0.090 *
	(0.049)
Health Status (Ref: Poor)	
Good	0.410 ***
	(0.029)
Fair	0.186***
	(0.034)
Regions (Ref: West)	
East	0.024
	(0.030)
Middle	−0.044 *
	(0.026)
Constant	2.205 ***
	(0.197)
Observations	6224
Log likelihood	−91114.406

Note: Standard errors in parentheses; *** *p* < 0.01, ** *p* < 0.05, * *p* < 0.1. Poor people: those whose income are below the average income.

**Table A2:** Mediation analysis (poor people).

Variables	Mediators		Dependent Variable
	Perceived Fairness	Trust	Happiness
Gini	−0.534 ***	−0.409 ***	−0.215
	(0.108)	(0.101)	(0.143)
Perceived Fairness			0.212 ***
			(0.010)
Trust			0.074 ***
			(0.010)
Income (CNY)			0.141 ***
			(0.014)
Sex (1 = Male)			−0.090 ***
			(0.021)
Age			−0.031 ***
			(0.004)
Age squared/100			0.033 ***
			(0.004)
Ethnicity (1 = Han)			−0.076 **
			(0.035)
Political status (1 = Party member)			0.084 **
			(0.035)
Marital Status (Ref: Others)			
Married			0.249 ***
			(0.035)
Single			0.018
			(0.059)
Household size			0.042 ***
			(0.007)
Residence (1 = Urban)			−0.043 *
			(0.024)
Work Status (Ref: Not working)			
Non-farm work			−0.035
			(0.028)
Farm work			−0.031
			(0.029)
Education degree (Ref: Primary education)			
Secondary education			0.049 **
			(0.024)
Tertiary education			0.079 *
			(0.046)
Health Status (Ref: Poor)			
Good			0.364 ***
			(0.028)
Fair			0.173 ***
			(0.032)
Regions (Ref: West)			
East			0.067 **
			(0.028)
Middle			−0.011
			(0.025)
Constant			1.445 ***
			(0.191)
Observations			6224
Log likelihood			−108,960.05

Note: Standard errors in parentheses; *** *p* < 0.01, ** *p* < 0.05, * *p* < 0.1.

**Table A3:** The impact of income inequality on happiness (rich people).

Variables	Happiness
Gini	−0.296 ***
	(0.113)
Income (CNY)	0.076 ***
	(0.028)
Sex (1 = Male)	−0.086 ***
	(0.028)
Age	−0.027 ***
	(0.006)
Age squared/100	0.031 ***
	(0.006)
Ethnicity (1 = Han)	−0.068
	(0.058)
Political status (1 = Party member)	0.067 *
	(0.034)
Marital Status (Ref: Others)	
Married	0.205 ***
	(0.047)
Single	−0.031
	(0.066)
Household size	0.082 ***
	(0.014)
Residence (1 = Urban)	−0.053
	(0.047)
Work Status (Ref: Not working)	
Non-farm work	−0.011
	(0.039)
Farm work	0.030
	(0.066)
Education degree (Ref: Primary education)	
Secondary education	−0.024
	(0.042)
Tertiary education	0.001
	(0.050)
Health Status (Ref: Poor)	
Good	0.452 ***
	(0.052)
Fair	0.202 ***
	(0.056)
Regions (Ref: West)	
East	0.005
	(0.043)
Middle	−0.096 **
	(0.047)
Constant	2.701 ***
	(0.346)
Observations	3015
Log likelihood	−39,570.63

Note: Standard errors in parentheses; *** *p* < 0.01, ** *p* < 0.05, * *p* < 0.1. Rich people: those whose income are above the average income.

**Table A4:** Mediation analysis (rich people).

Variables	Mediators		Dependent Variable
	Perceived Fairness	Trust	Happiness
Gini	−0.308 ***	−0.687 ***	−0.192
	(0.107)	(0.159)	(0.128)
Perceived Fairness			0.137 ***
			(0.013)
Trust			0.090 ***
			(0.013)
Income (CNY)			0.067 **
			(0.027)
Sex (1 = Male)			−0.079 ***
			(0.027)
Age			−0.025 ***
			(0.006)
Age squared/100			0.027 ***
			(0.005)
Ethnicity (1 = Han)			−0.031
			(0.056)
Political status (1 = Party member)			0.052
			(0.033)
Marital Status (Ref: Others)			
Married			0.203 ***
			(0.045)
Single			−0.030
			(0.064)
Household size			0.076 ***
			(0.013)
Residence (1 = Urban)			0.003
			(0.045)
Work Status (Ref: Not working)			
Non-farm work			−0.022
			(0.037)
Farm work			−0.017
			(0.064)
Education degree (Ref: Primary education)			
Secondary education			−0.005
			(0.041)
Tertiary education			−0.004
			(0.049)
Health Status (Ref: Poor)			
Good			0.412 ***
			(0.050)
Fair			0.198 ***
			(0.055)
Regions (Ref: West)			
East			0.007
			(0.042)
Middle			−0.100 **
			(0.046)
Constant			2.054 ***
			(0.337)
Observations			3015
Log likelihood			−48,180.501

Note: Standard errors in parentheses; *** *p* < 0.01, ** *p* < 0.05.

**Table A5:** The impact of income inequality on happiness(urban).

Variables	Happiness
Gini	−0.166 **
	(0.081)
Income (CNY)	0.140 ***
	(0.014)
Sex (1 = Male)	−0.112 ***
	(0.022)
Age	−0.031 ***
	(0.005)
Age squared/100	0.033 ***
	(0.004)
Ethnicity (1 = Han)	−0.104 **
	(0.046)
Political status (1 = Party member)	0.071 **
	(0.029)
Marital Status (Ref: Others)	
Married	0.195 ***
	(0.037)
Single	−0.082
	(0.055)
Household size	0.059 ***
	(0.009)
Work Status (Ref: Not working)	
Non-farm work	−0.074 ***
	(0.028)
Farm work	0.094 *
	(0.057)
Education degree (Ref: Primary education)	
Secondary education	0.004
	(0.030)
Tertiary education	0.048
	(0.039)
Health Status (Ref: Poor)	
Good	0.441 ***
	(0.036)
Fair	0.198 ***
	(0.039)
Regions (Ref: West)	
East	0.031
	(0.032)
Middle	0.008
	(0.032)
Constant	2.228 ***
	(0.197)
Observations	5635
Log likelihood	−77,053.037

Note: Standard errors in parentheses; *** *p* < 0.01, ** *p* < 0.05, * *p* < 0.1.

**Table A6:** Mediation analysis (urban).

Variables	Mediators		Dependent Variable
	Perceived Fairness	Trust	Happiness
Gini	−0.173 **	−0.351 **	−0.102
	(0.086)	(0.140)	(0.101)
Perceived Fairness			0.163 ***
			(0.010)
Trust			0.097 ***
			(0.010)
Income (CNY)			0.132 ***
			(0.013)
Sex (1 = Male)			−0.101 ***
			(0.021)
Age			−0.028 ***
			(0.004)
Age squared/100			0.029 ***
			(0.004)
Ethnicity (1 = Han)			−0.086 *
			(0.044)
Political status (1 = Party member)			0.064 **
			(0.028)
Marital Status (Ref: Others)			
Married			0.204 ***
			(0.035)
Single			−0.069
			(0.053)
Household size			0.056 ***
			(0.009)
Work Status (Ref: Not working)			
Non-farm work			−0.083 ***
			(0.027)
Farm work			0.054
			(0.054)
Education degree (Ref: Primary education)			
Secondary education			0.018
			(0.029)
Tertiary education			0.039
			(0.038)
Health Status (Ref: Poor)			
Good			0.382 ***
			(0.035)
Fair			0.176 ***
			(0.038)
Regions (Ref: West)			
East			0.053 *
			(0.031)
Middle			0.014
			(0.031)
Constant			1.504 ***
			(0.192)
Observations			5635
Log likelihood			−93,272.896

Note: Standard errors in parentheses; *** *p* < 0.01, ** *p* < 0.05, * *p* < 0.1.

**Table A7:** The impact of income inequality on happiness(rural).

Variables	Happiness
Gini	−0.454 ***
	(0.101)
Income (CNY)	0.145 ***
	(0.014)
Sex (1 = Male)	−0.072 **
	(0.029)
Age	−0.031 ***
	(0.006)
Age squared/100	0.038 ***
	(0.006)
Ethnicity (1 = Han)	−0.068
	(0.042)
Political status (1 = Party member)	0.153 ***
	(0.053)
Marital Status (Ref: Others)	
Married	0.291 ***
	(0.048)
Single	0.089
	(0.081)
Household size	0.038 ***
	(0.010)
Work Status (Ref: Not working)	
Non-farm work	0.067
	(0.044)
Farm work	0.032
	(0.035)
Education degree (Ref: Primary education)	
Secondary education	0.054 *
	(0.031)
Tertiary education	0.157 *
	(0.092)
Health Status (Ref: Poor)	
Good	0.395 ***
	(0.036)
Fair	0.183 ***
	(0.043)
Regions (Ref: West)	
East	0.067 *
	(0.038)
Middle	−0.112 ***
	(0.031)
Constant	2.304 ***
	(0.246)
Observations	3604
Log likelihood	−48,658.478

Note: Standard errors in parentheses; *** *p* < 0.01, ** *p* < 0.05, * *p* < 0.1.

**Table A8:** Mediation analysis (rural).

Variables	Mediators		Dependent Variable
	Perceived Fairness	Trust	Happiness
Gini	−0.861 ***	−0.193 ***	−0.248
	(0.250)	(0.006)	(0.194)
Perceived Fairness			0.229 ***
			(0.013)
Trust			0.046 ***
			(0.013)
Income (CNY)			0.135 ***
			(0.014)
Sex (1 = Male)			−0.064 **
			(0.027)
Age			−0.034 ***
			(0.006)
Age squared/100			0.038 ***
			(0.005)
Ethnicity (1 = Han)			−0.066 *
			(0.040)
Political status (1 = Party member)			0.104 **
			(0.051)
Marital Status (Ref: Others)			
Married			0.297 ***
			(0.045)
Single			0.103
			(0.077)
Household size			0.038 ***
			(0.009)
Work Status (Ref: Not working)			
Non-farm work			0.078 *
			(0.041)
Farm work			0.012
			(0.033)
Education degree (Ref: Primary education)			
Secondary education			0.057 *
			(0.030)
Tertiary education			0.135
			(0.088)
Health Status (Ref: Poor)			
Good			0.358 ***
			(0.034)
Fair			0.175 ***
			(0.040)
Regions (Ref: West)			
East			0.104 ***
			(0.037)
Middle			−0.067 **
			(0.030)
Constant			1.751 ***
			(0.236)
Observations			3604
Log likelihood			−58,763.191

Note: Standard errors in parentheses; *** *p* < 0.01, ** *p* < 0.05, * *p* < 0.1.

**Table A9:** The impact of income inequality on happiness (young people).

Variables	Happiness
Gini	−0.312 ***
	(0.156)
Income (CNY)	0.167 ***
	(0.013)
Sex (1 = Male)	−0.059 ***
	(0.020)
Age	−0.048 ***
	(0.009)
Age squared/100	0.055 ***
	(0.010)
Ethnicity (1 = Han)	−0.092 ***
	(0.035)
Political status (1 = Party member)	0.069 **
	(0.030)
Marital Status (Ref: Others)	
Married	0.394 ***
	(0.044)
Single	0.104 *
	(0.058)
Household size	0.056 ***
	(0.009)
Residence (1 = Urban)	−0.121 ***
	(0.026)
Work Status (Ref: Not working)	
Non-farm work	−0.034
	(0.027)
Farm work	−0.024
	(0.034)
Education degree (Ref: Primary education)	
Secondary education	0.071 ***
	(0.026)
Tertiary education	0.106 ***
	(0.037)
Health Status (Ref: Poor)	
Good	0.434 ***
	(0.034)
Fair	0.200 ***
	(0.039)
Regions (Ref: West)	
East	0.029
	(0.028)
Middle	−0.035
	(0.026)
Constant	2.140 ***
	(0.230)
Observations	6515
Log likelihood	−85,327.168

Note: Standard errors in parentheses; *** *p* < 0.01, ** *p* < 0.05, * *p* < 0.1. Young people: those who are below 60 years old.

**Table A10:** Mediation analysis (young people).

Variables	Mediators		Dependent Variable
	Perceived Fairness	Trust	Happiness
Gini	−0.525 ***	−0.413 ***	−0.196
	(0.107)	(0.123)	(0.121)
Perceived Fairness			0.158 ***
			(0.009)
Trust			0.079 ***
			(0.009)
Income (CNY)			0.158 ***
			(0.012)
Sex (1 = Male)			−0.057 ***
			(0.020)
Age			−0.046 ***
			(0.008)
Age squared/100			0.052 ***
			(0.010)
Ethnicity (1 = Han)			−0.088 ***
			(0.034)
Political status (1 = Party member)			0.044
			(0.029)
Marital Status (Ref: Others)			
Married			0.374 ***
			(0.042)
Single			0.099 *
			(0.056)
Household size			0.055 ***
			(0.008)
Residence (1 = Urban)			−0.069 ***
			(0.025)
Work Status (Ref: Not working)			
Non-farm work			−0.040
			(0.026)
Farm work			−0.063 *
			(0.033)
Education degree (Ref: Primary education)			
Secondary education			0.080 ***
			(0.025)
Tertiary education			0.094 ***
			(0.036)
Health Status (Ref: Poor)			
Good			0.389 ***
			(0.033)
Fair			0.192 ***
			(0.038)
Regions (Ref: West)			
East			0.047 *
			(0.027)
Middle			−0.019
			(0.025)
Constant			1.506 ***
			(0.224)
Observations			6515
Log likelihood			−104,054.42

Note: Standard errors in parentheses; *** *p* < 0.01, * *p* < 0.1.

**Table A11:** The impact of income inequality on happiness (old people).

Variables	Happiness
Gini	−0.304 **
	(0.142)
Income (CNY)	0.096 ***
	(0.016)
Sex (1 = Male)	−0.165 ***
	(0.034)
Age	0.077 **
	(0.038)
Age squared/100	−0.046 *
	(0.026)
Ethnicity (1 = Han)	−0.061
	(0.062)
Political status (1 = Party member)	0.145 ***
	(0.048)
Marital Status (Ref: Others)	
Married	0.128 ***
	(0.040)
Single	−0.360 ***
	(0.138)
Household size	0.043 ***
	(0.011)
Residence (1 = Urban)	−0.025
	(0.042)
Work Status (Ref: Not working)	
Non-farm work	−0.016
	(0.059)
Farm work	0.051
	(0.046)
Education degree (Ref: Primary education)	
Secondary education	−0.030
	(0.039)
Tertiary education	−0.086
	(0.076)
Health Status (Ref: Poor)	
Good	0.421 ***
	(0.039)
Fair	0.191 ***
	(0.043)
Regions (Ref: West)	
East	−0.035
	(0.047)
Middle	−0.113 ***
	(0.043)
Constant	−0.857
	(1.364)
Observations	2724
Log likelihood	−33,617.62

Note: Standard errors in parentheses; *** *p* < 0.01, ** *p* < 0.05, * *p* < 0.1. Old people: those who are above 60 years old.

**Table A12:** Mediation analysis (old people).

Variables	Mediators		Dependent Variable
	Perceived Fairness	Trust	Happiness
Gini	−0.196 ***	−0.687 ***	−0.202
	(0.201)	(0.149)	(0.177)
Perceived Fairness			0.252 ***
			(0.015)
Trust			0.076 ***
			(0.015)
Income (CNY)			0.089 ***
			(0.015)
Sex (1 = Male)			−0.132 ***
			(0.032)
Age			0.059 *
			(0.035)
Age squared/100			−0.035
			(0.024)
Ethnicity (1 = Han)			−0.013
			(0.058)
Political status (1 = Party member)			0.151 ***
			(0.045)
Marital Status (Ref: Others)			
Married			0.154 ***
			(0.038)
Single			−0.436 ***
			(0.130)
Household size			0.041 ***
			(0.010)
Residence (1 = Urban)			0.072 *
			(0.040)
Work Status (Ref: Not working)			
Non-farm work			−0.029
			(0.055)
Farm work			0.034
			(0.043)
Education degree (Ref: Primary education)			
Secondary education			−0.013
			(0.037)
Tertiary education			−0.089
			(0.071)
Health Status (Ref: Poor)			
Good			0.373 ***
			(0.037)
Fair			0.162 ***
			(0.041)
Regions (Ref: West)			
East			0.033
			(0.044)
Middle			−0.060
			(0.040)
Constant			−1.183
			(1.279)
Observations			2724
Log likelihood			−41,258.739

Note: Standard errors in parentheses; *** *p* < 0.01, * *p* < 0.1.

**Table A13:** The impact of income inequality on happiness (male).

Variables	Happiness
Gini	−0.344 ***
	(0.115)
Income (CNY)	0.142 ***
	(0.014)
Age	−0.034 ***
	(0.005)
Age squared/100	0.038 ***
	(0.005)
Ethnicity (1 = Han)	−0.031
	(0.043)
Political status (1 = Party member)	0.120 ***
	(0.031)
Marital Status (Ref: Others)	
Married	0.188 ***
	(0.044)
Single	−0.090
	(0.061)
Household size	0.049 ***
	(0.009)
Residence (1 = Urban)	−0.151 ***
	(0.031)
Work Status (Ref: Not working)	
Non-farm work	0.056
	(0.035)
Farm work	0.058
	(0.040)
Education degree (Ref: Primary education)	
Secondary education	0.029
	(0.030)
Tertiary education	0.035
	(0.045)
Health Status (Ref: Poor)	
Good	0.467 ***
	(0.036)
Fair	0.218 ***
	(0.041)
Regions (Ref: West)	
East	0.002
	(0.034)
Middle	−0.055 *
	(0.031)
Constant	2.205 ***
	(0.210)
Observations	4787
Log likelihood	−68,520.689

Note: Standard errors in parentheses; *** *p* < 0.01, * *p* < 0.1.

**Table A14:** Mediation analysis (male).

Variables	Mediators		Dependent Variable
	Perceived Fairness	Trust	Happiness
Gini	−0.524 ***	−0.474 ***	−0.201
	(0.142)	(0.145)	(0.119)
Perceived Fairness			0.196 ***
			(0.011)
Trust			0.087 ***
			(0.011)
Income (CNY)			0.137 ***
			(0.013)
Age			−0.033 ***
			(0.005)
Age squared/100			0.035 ***
			(0.005)
Ethnicity (1 = Han)			−0.013
			(0.041)
Political status (1 = Party member)			0.099 ***
			(0.030)
Marital Status (Ref: Others)			
Married			0.184 ***
			(0.042)
Single			−0.094
			(0.058)
Household size			0.048 ***
			(0.009)
Residence (1 = Urban)			−0.074 **
			(0.030)
Work Status (Ref: Not working)			
Non-farm work			0.044
			(0.033)
Farm work			0.015
			(0.038)
Education degree (Ref: Primary education)			
Secondary education			0.031
			(0.029)
Tertiary education			0.001
			(0.043)
Health Status (Ref: Poor)			
Good			0.424 ***
			(0.034)
Fair			0.226 ***
			(0.039)
Regions (Ref: West)			
East			0.031
			(0.032)
Middle			−0.029
			(0.029)
Constant			1.390 ***
			(0.203)
Observations			4787
Log likelihood			−82,293.409

Note: Standard errors in parentheses; *** *p* < 0.01, ** *p* < 0.05.

**Table A15:** The impact of income inequality on happiness (female).

Variables	Happiness
Gini	0.291 ***
	(0.101)
Income (CNY)	0.128 ***
	(0.014)
Age	−0.028 ***
	(0.005)
Age squared/100	0.035 ***
	(0.005)
Ethnicity (1 = Han)	−0.113 **
	(0.044)
Political status (1 = Party member)	0.035
	(0.045)
Marital Status (Ref: Others)	
Married	0.294 ***
	(0.039)
Single	0.099
	(0.070)
Household size	0.048 ***
	(0.010)
Residence (1 = Urban)	−0.044
	(0.032)
Work Status (Ref: Not working)	
Non-farm work	−0.082 ***
	(0.032)
Farm work	−0.036
	(0.037)
Education degree (Ref: Primary education)	
Secondary education	0.035
	(0.031)
Tertiary education	0.119 **
	(0.049)
Health Status (Ref: Poor)	
Good	0.377 ***
	(0.035)
Fair	0.156 ***
	(0.040)
Regions (Ref: West)	
East	0.037
	(0.035)
Middle	−0.043
	(0.032)
Constant	2.221 ***
	(0.209)
Observations	4452
Log likelihood	−62,345.294

Note: Standard errors in parentheses; *** *p* < 0.01, ** *p* < 0.05.

**Table A16:** Mediation analysis (female).

	Mediators		Dependent Variable
Variables	Perceived Fairness	Trust	Happiness
Gini	−0.521 ***	−0.368 ***	−0.171
	(0.155)	(0.155)	(0.130)
Perceived Fairness			0.180 ***
			(0.012)
Trust			0.067 ***
			(0.012)
Income (CNY)			0.120 ***
			(0.013)
Age			−0.027 ***
			(0.005)
Age squared/100			0.031 ***
			(0.005)
Ethnicity (1 = Han)			−0.107 **
			(0.043)
Political status (1 = Party member)			0.023
			(0.043)
Marital Status (Ref: Others)			
Married			0.302 ***
			(0.038)
Single			0.114 *
			(0.068)
Household size			0.048 ***
			(0.009)
Residence (1 = Urban)			0.012
			(0.031)
Work Status (Ref: Not working)			
Non-farm work			−0.095 ***
			(0.030)
Farm work			−0.062 *
			(0.036)
Education degree (Ref: Primary education)			
Secondary education			0.060 **
			(0.030)
Tertiary education			0.128 ***
			(0.047)
Health Status (Ref: Poor)			
Good			0.333 ***
			(0.034)
Fair			0.132 ***
			(0.038)
Regions (Ref: West)			
East			0.065 *
			(0.033)
Middle			−0.022
			(0.031)
Constant			1.527 ***
			(0.205)
Observations			4452
Log likelihood			−75,048.271

Note: Standard errors in parentheses; *** *p* < 0.01, ** *p* < 0.05, * *p* < 0.1.
