# Supplementary material for: Psychological Mechanisms Linking County-Level Income Inequality to Happiness in China
Source: Int J Environ Res Public Health. 2018 Nov 27;15(12):2667. doi: 10.3390/ijerph15122667 (PMC6313330; doi:10.3390/ijerph15122667)
Supplement: Supplementary file 1 [file ijerph-15-02667-s001.pdf]

Additional file 1: robust test

Exclude counties with no more than 10 observations.

Table S1. The impact of income inequality on happiness.

Variables	Happiness
Gini	-0.307**
	(0.140)
Income	0.136***
	(0.010)
Sex (1=Male)	-0.096***
	(0.018)
Age	-0.031***
	(0.004)
Age squared /100	0.036***
	(0.004)
Ethnicity (1=Han)	-0.076**
	(0.031)
Political status (1=Party member)	0.088***
	(0.027)
Marital Status (Ref: Others)	
Married	0.233***
	(0.030)
Single	-0.011
	(0.047)
Household size	0.047***
	(0.007)
Residence (1=Urban)	-0.100***
	(0.023)
Work Status (Ref: Not working)	
Non-farm work	-0.020
	(0.024)
Farm work	-0.003
	(0.028)
Education degree (Ref: Primary education)	
Secondary education	0.037
	(0.023)
Tertiary education	0.063*
	(0.035)
Health Status (Ref: Poor)	

Good	0.419***
	(0.026)
Fair	0.179***
	(0.030)
Regions (Ref: West)	
East	0.019
	(0.025)
Middle	-0.055**
	(0.023)
Constant	2.300***
	(0.156)
Observations	8,605
Log likelihood	-129632.53

Note: Standard errors in parentheses; *** p<0.01, ** p<0.05, * p<0.1

Table S2. Mediation analysis.

Variables	Mediators		Dependent variable
	Perceived Fairness	Trust	Happiness
Gini	-0.522***	-0.428***	-0.175
	(0.108)	(0.106)	(0.141)
Perceived Fairness			0.188***
			(0.008)
Trust			0.078***
			(0.008)
Income			0.130***
			(0.010)
Sex (1=Male)			-0.086***
			(0.018)
Age			-0.030***
			(0.004)
Age squared /100			0.033***
			(0.003)
Ethnicity (1=Han)			-0.060**
			(0.030)
Political status (1=Party member)			0.068***
			(0.026)
Marital Status (Ref: Others)			
Married			0.237***

	(0.029)
Single	-0.004
	(0.045)
Household size	0.048***
	(0.007)
Residence (1=Urban)	-0.032
	(0.022)
Work Status (Ref: Not working)	
Non-farm work	-0.027
	(0.023)
Farm work	-0.031
	(0.027)
Education degree (Ref: Primary education)	
Secondary education	0.052**
	(0.022)
Tertiary education	0.054
	(0.033)
Health Status (Ref: Poor)	
Good	0.376***
	(0.025)
Fair	0.167***
	(0.028)
Regions (Ref: West)	
East	0.050**
	(0.024)
Middle	-0.023
	(0.022)
Constant	1.502***
	(0.152)
Observations	8605
Log likelihood	-154419.11

Note: Standard errors in parentheses; *** p<0.01, ** p<0.05, * p<0.1

Exclude counties with no more than 20 observations.

Table S3. The impact of income inequality on happiness.

Variables	Happiness
Gini	-0.310**
	(0.148)
Income	0.137***
	(0.011)
Sex (1=Male)	-0.104***
	(0.019)
Age	-0.031***
	(0.004)
Age squared /100	0.036***
	(0.004)
Ethnicity (1=Han)	-0.055*
	(0.032)
Political status (1=Party member)	0.073***
	(0.028)
Marital Status (Ref: Others)	
Married	0.255***
	(0.032)
Single	0.001
	(0.050)
Household size	0.045***
	(0.007)
Residence (1=Urban)	-0.103***
	(0.024)
Work Status (Ref: Not working)	
Non-farm work	-0.026
	(0.025)
Farm work	0.001
	(0.029)
Education degree (Ref: Primary education)	
Secondary education	0.048**
	(0.023)
Tertiary education	0.083**
	(0.036)
Health Status (Ref: Poor)	
Good	0.409***
	(0.027)
Fair	0.179***
	(0.031)
Regions (Ref: West)	
East	0.039

	(0.026)
Middle	-0.044*
	(0.024)
Constant	2.238***
	(0.161)
Observations	7,935
Log likelihood	-119711.84

Note: Standard errors in parentheses; *** p<0.01, ** p<0.05, * p<0.1

Table S4. Mediation analysis.

Variables	Mediators		Dependent variable
	Perceived Fairness	Trust	Happiness
Gini	-0.543***	-0.429***	-0.170
	(0.113)	(0.116)	(0.138)
Perceived Fairness			0.196***
			(0.009)
Trust			0.076***
			(0.009)
Income			0.134***
			(0.010)
Sex (1=Male)			-0.092***
			(0.018)
Age			-0.030***
			(0.004)
Age squared /100			0.033***
			(0.004)
Ethnicity (1=Han)			-0.042
			(0.031)
Political status (1=Party member)			0.050*
			(0.027)
Marital Status (Ref: Others)			
Married			0.257***
			(0.030)
Single			0.008
			(0.047)
Household size			0.046***
			(0.007)
Residence (1=Urban)			-0.036
			(0.023)
Work Status (Ref: Not working)			
Non-farm work			-0.032
			(0.024)
Farm work			-0.025

	(0.028)
Education degree (Ref: Primary education)	
Secondary education	0.060***
	(0.022)
Tertiary education	0.073**
	(0.035)
Health Status (Ref: Poor)	
Good	0.360***
	(0.026)
Fair	0.161***
	(0.029)
Regions (Ref: West)	
East	0.068***
	(0.025)
Middle	-0.017
	(0.023)
Constant	1.433***
	(0.157)
Observations	7935
Log likelihood	-142577.16

Note: Standard errors in parentheses; *** p<0.01, ** p<0.05, * p<0.1
